# Detection and quantification of pestivirus in experimentally infected pregnant ewes and their progeny

**DOI:** 10.1186/1743-422X-6-189

**Published:** 2009-11-05

**Authors:** Ana Hurtado, Isbene Sanchez, Felix Bastida, Esmeralda Minguijón, Ramón A Juste, Ana L García-Pérez

**Affiliations:** 1 NEIKER - Instituto Vasco de Investigación y Desarrollo Agrario, Department of Animal Health, Berreaga 1, 48160 Derio, Bizkaia, Spain; 2 Vacunek SL, Ibaizabal Bidea 800, 48160 Derio, Bizkaia, Spain

## Abstract

**Background:**

Border disease virus (BDV) causes important reproductive losses, and eradication strategies focus on the identification and removal of persistently infected animals arising after in uterine infection. BDV infection dynamics were studied in 13 ewes experimentally infected with BDV-4 genotype at 3 phases of pregnancy [days 108 (group A), 76 (group B) and 55 (group C)] by quantification of viral RNA in blood collected on days -1 to parturition using quantitative real-time RT-PCR (qRT-PCR). Viral RNA loads were also measured in blood/foetal fluid and tissue samples from their offspring at lambing (3 foetuses, 7 stillborns, 15 lambs). qRT-PCR results were compared with those obtained by conventional RT-PCR and used to predict persistent infections.

**Results:**

Viral RNA was detected in the ewes between days 2-15 p.i. The viraemia reached its highest peak between days 6-7 p.i. with a second peak at days 11-12 p.i. qRT-PCR was significantly faster to perform (less than 1 h) than conventional RT-PCR and detected BDV RNA in more ewes, being detection more continuous and prolonged in time. The virus was detected in peripheral blood in a higher percentage of lambs than in tissues, where differences in viral genome copies were more marked. Skin and cerebral cortex showed the highest viral RNA loads, and spleen and spinal cord the lowest. High viral RNA loads were observed in several animals in group B and all in group C, infected during middle and early foetal development, respectively, but also in one lamb from group A, infected during late foetal development. Serology and viral genome copy number estimates in blood and tissues were used to establish a quantitative cut-off threshold for transient viraemia.

**Conclusion:**

Viral RNA quantification showed potential for the discrimination between persistent infections and transient viraemia using single-time point blood sampling and raised questions regarding foetal immune system development and the occurrence of persistent infections.

## Background

The genus Pestivirus (family Flaviviridae) comprises four main species: bovine viral diarrhoea virus types 1 and 2 (BVDV 1 and BVDV 2), border disease virus (BDV) of sheep, and classical swine fever virus (CSFV), each of them subdivided into several genetic subtypes. In sheep, infection of pregnant ewes with BDV during early or mid gestation results in abortion, stillbirths, or unviable lambs. Infections during early embryonic and foetal development can lead to the birth of immunotolerant and seronegative persistently infected (PI) animals that shed the virus throughout their lifetime and are the continuous source of infection within and among flocks. Border disease (BD) has been reported in several regions in Spain [1,2], and it is widely spread in the Basque Country (Northern Spain) [3] where it is considered one of the main causes of ovine abortion [4]. Unfortunately, there are no commercial vaccines available for small ruminants. Effective control measures are based on identifying and eliminating PI animals, and therefore, reliable diagnostic techniques are essential for detecting the presence of the virus and for investigating biological aspects of the infection like dynamics, transmission or viral load.

Pestiviruses are small enveloped viruses with a genome consisting of a positive-sense single stranded RNA molecule of approximately 12.3 kb. It is comprised of a long single open reading frame flanked by untranslated regions (UTR) of about 380 nucleotides at the 5'-end, and 230 nts at the 3'-end [5,6]. The 5'-UTR includes two highly conserved regions approximately 250 nucleotides apart, which allow for the design of primers capable of detecting a wide range of pestiviruses. The 5'-UTR is therefore a very convenient target for rapid detection of unknown pestivirus isolates by reverse transcription polymerase chain reaction (RT-PCR), a technique routinely used for pestivirus diagnosis in blood and tissue samples since the nineties. RT-PCR has shown a better performance than antigen-ELISA in detecting transient viraemia in blood from experimentally infected sheep [7], as well as in blood or foetal fluids from new born lambs and stillborns [8]. However, conventional RT-PCR is being replaced by real-time RT-PCR, a technique that permits quantitative detection of the target, providing an estimate of the viral RNA load in infected animals. In addition, real-time PCR eliminates post-amplification processing of the products reducing the chances of carryover contamination and speeding up the process. Furthermore, were it capable of differentiating between animals with transient viraemia and persistent infections, quantification could provide critical information for pestiviral control programmes.

In this article, we describe the performance of a real-time RT-PCR assay for the detection and quantification of pestiviruses on different types of samples obtained from an experimental infection carried out on pregnant ewes challenged with the local ovine pestivirus (BDV-4 genotype) in different gestation periods as described elsewhere [7]. BDV infection dynamics were studied in the experimentally infected ewes, and viral RNA loads were measured in blood or foetal fluid and tissue samples from their offspring at lambing.

## Methods

### Experiment

Samples were obtained from an experimental infection carried out on pregnant ewes as described elsewhere [7]. Briefly, 13 virus- and antibody-negative, artificially inseminated pregnant ewes were challenged at different stages of gestation on days 108 (group A, 5 ewes), 76 (group B, 5 ewes) or 55 (group C, 3 ewes) with an ovine pestivirus (BDV-4 genotype, strain 0502234, GenBank Acc. No. EU711348). The outcomes of pregnancy included 3 aborted foetuses, 7 stillborns and 15 lambs.

### Samples

In experimental ewes, blood samples were collected daily during the first two weeks and weekly until lambing, adding up to a total of 293 blood samples. Regarding the offspring, pre-colostral blood samples were taken from live lambs just after lambing, and cardiac blood or foetal fluids were collected from stillborns and foetuses. Presence of BDV antibodies in the offspring as determined by ELISA had been reported elsewhere [7]. Live lambs were euthanized within 24 hr of birth. At necropsy, tissue samples (brain [cerebral frontal cortex, mesencephalon, cerebellum], spleen, kidney, thyroid gland, thymus, spinal cord, lymph node, and skin) were collected and preserved either at -80°C or at -20°C submerged in a RNA stabilization reagent (RNAlater™ RNA Stabilization Reagent, Qiagen). Fifteen placenta samples were also collected. Hence, a total of 243 tissue samples were included in the study.

### Virus detection and quantification by real-time RT-PCR

RNA was extracted with a QIAamp Viral RNA Mini kit (Qiagen) as previously described [8]. Blood and tissue samples previously analysed by a one-tube RT-PCR [7,8] were subjected to a commercially available quantitative real-time RT-PCR (qRT-PCR) assay for the detection and quantification of pestiviruses (BehiBVD/BD-VK, Vacunek, S.L.). This is a duplex assay that includes a TaqMan LNA probe targeting the 5'-UTR of the different genotypes, and a probe that detects a host-encoded gene used as internal control. Reactions were run in an ABI Prism 7500 Sequence Detection System (Applied Biosystems) in a 25 μl volume consisting of 22.5 μl of BehiBVD/BD-VK Master Mix and 2.5 μl of RNA, using the following program: 5 min at 42°C, 10 s at 95°C and 40 cycles of 5 s at 95°C and 34 s at 60°C.

Detection capacity of the kit assessed by bioinformatic analysis of all the pestivirus sequences available in GenBank as of May 2009 indicated that the primers and probe used would recognise 99.9% of the pestivirus isolates. Furthermore, a panel of pestiviruses of different types including CSFV (strain Alfort, genotype CSFV 1.1, and Brescia, CSFV 1.2.), BDV (clinical isolate, genotype 4) and BVDV (clinical isolates, genotypes 1b and 1e) was empirically tested. The analytical specificity was evaluated using RNA from Tick-borne encephalitis virus, Spanish sheep encephalitis virus, bluetongue virus, influenza virus, maedi-visna virus and pulmonary adenomatosis virus. The linearity and analytical sensitivity of the real-time RT-PCR quantification protocol were assessed on 10-fold serial dilutions of a recombinant RNA standard (synthesised in vitro from a cloned RT-PCR fragment in plasmid DNA). For viral RNA load quantification, samples were analysed in triplicate, and each plate contained six to eight ten serial dilutions (10^8^-10 copies) of the RNA positive control in triplicate for the standard curve. The efficiency of the real-time PCR amplification (E) was calculated for each experiment using the formula E = 10^-1/m^, where m is the slope for the standard curve. Three non-template negative controls were also included in each plate.

### Statistical analysis

To verify inter-assay reproducibility, analysis of variance of the Ct values obtained for the standard curves was carried out with the General Linear Models (GLM) procedure of the SAS statistical package version 9.1 (SAS institute, Cary, NC, USA). In addition, comparison of the slopes was performed using the CONTRAST statement of the GLM procedure in the SAS statistical package.

The level of agreement between methods (conventional RT-PCR performed on the same samples elsewhere [7,8] and quantitative real-time RT-PCR as reported herein) was tested by the Kappa index test at a 95% confidence interval using Win Episcope 2.0 and the degree of correspondence (relative accuracy) was calculated as 100 × (no. samples positive and negative by both methods/total no. samples analysed by both methods). A variable called complementary sensitivity (CS) of one method over the other was calculated using the following formula: 100 × (no. samples positive by method 1 and negative by method 2/total no. samples positive by method 2). It measures the additional detection efficacy of method 1 over method 2 when both have similar specificity [9].

Differences in viral genome copy number between ewes and lambs groups were tested with GLM procedure of the SAS statistical package. A *P *value of less than 0.05 was considered significant.

## Results

### Specificity and sensitivity of the qRT-PCR assay

The qRT-PCR kit successfully amplified all the pestiviruses tested (CSFV, BDV, BVDV), but none of the non-pestiviral RNA samples. In spite of the reduced number of viruses used, neither the flaviviruses (Tick-borne encephalitis virus, Spanish sheep encephalitis virus) nor other common viruses of sheep (bluetongue virus, maedi-visna virus, pulmonary adenomatosis virus) used to test the analytical specificity, showed any cross-reaction.

The amplification efficiency reached 1.91 and the correlation coefficient (R^2^) was 0.998. Quantification was linear over at least 7 log units and the analytical sensitivity was set at 10 copies/reaction. The analyses of variance of the Ct values of the standard curves showed no replicate nor plate effect (p > 0.25). Similarly, the slopes of the standard curves from the different plates did not significantly differ (p > 0.40), demonstrating a good reproducibility from run to run.

Parallel analysis of the results obtained herein and those previously reported using a conventional RT-PCR on the same samples [7,8] showed a good level of agreement between methods (Kappa = 0.702) and 86.3% of relative accuracy. Overall, the diagnostic sensitivity of qRT-PCR was significantly higher, as indicated by the 45.6% CS of qRT-PCR over conventional RT-PCR. Particularly significant was the improvement observed for blood samples collected from ewes (CS = 79.1%), where real-time qRT-PCR detected BDV in 34 samples negative by conventional PCR and provided a more continuous and prolonged detection in time. In tissues, the most significant improvement was observed for mesencephalon (CS = 150%) and cortex and thymus (CS = 71.4%).

### Dynamics of BDV in infected ewes

**Figure 1:**
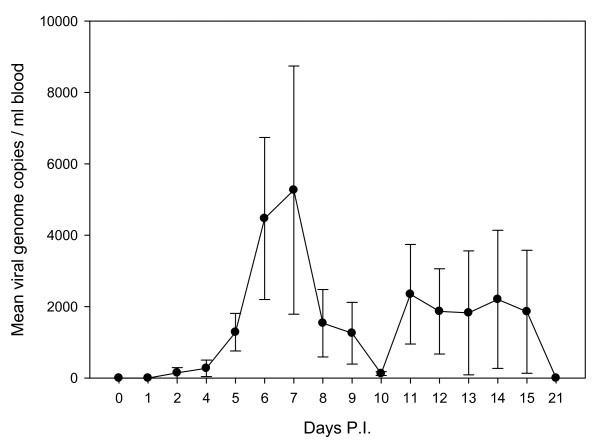
**Dynamics and viral RNA loads of BDV infection in blood from ewes experimentally infected with an ovine pestivirus (BDV-4 genotype)**. Line Plot represents mean viral genome copies per ml of blood at different days post-infection. Error bars above and below the line indicate the standard error of the mean.

In the experimentally infected ewes, viral RNA could be detected in blood by qRT-PCR as early as day 2 p.i. in one ewe. Between days 6-7 p.i. the viraemia reached its highest peak and then decreased to increase again at days 11-12 p.i., being all the ewes negative by day 21 p.i. (Fig. 1). The longest viraemia was observed in a ewe from group B which was BDV RNA-positive from day 2 to 15 post-challenge. One ewe of group A remained negative throughout the study. The estimates for viral genome copy number ranged from 0 to 42,430 per ml of blood.

### Quantification of BDV in the progeny

**Figure 2:**
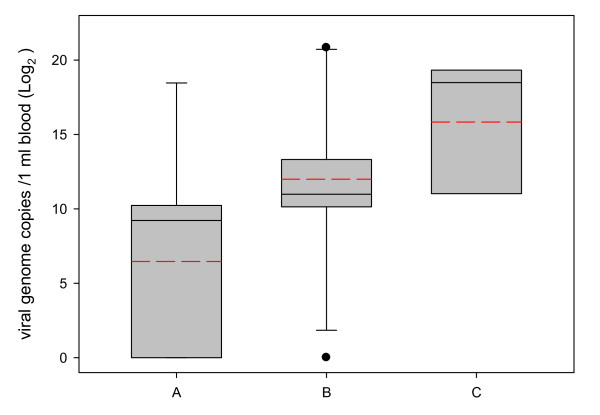
**Box Plot representing log_2 _viral genome copies per ml of blood in the offspring grouped according to the time of infection**. Group A, offspring of ewes challenged at day 108 of gestation; group B, at day 76; and, group C, at day 55. The boundary of the box closest to zero indicates the 25th percentile, the continuous line within the box marks the median, the dashed line marks the mean and the boundary of the box farthest from zero indicates the 75th percentile. Error bars above and below the box indicate the 90th and 10th percentiles. Outlying points are represented as closed dots.

Viral RNA was detected in blood or foetal fluid samples from 20/22 of the lambs and stillborns, including 5 animals that had tested negative by conventional RT-PCR [8]. Foetal fluids from the 3 foetuses were all negative, despite lack of inhibition, as confirmed by the amplification of the internal control. The mean viral genome copy number in blood for live lambs from group A (60,775 copies/ml blood) was marginally lower (*P *= 0.07) than that observed in lambs from group C (510,784), but no statistical differences were observed between lambs from groups A and B or B and C (Fig. 2). When considering the prenatal humoral response, an inverse association was found between viraemia and the presence of non-colostral antibodies determined by ELISA. This was clearly observed in the offspring from ewes in group C, which had high genome copy numbers in blood and no antibodies (Table 1). A high number of viral genome copies/ml blood in the absence of antibodies was also observed in two lambs from group B (Lambs No. 12 and 20) and one from group A (Lamb No. 4).

**Table 1:** List of ewes experimentally infected with pestivirus (BDV-genotype 4) along with their progeny and a summary of qualitative and quantitative results of qRT-PCR in placentae, and blood and tissues from the offspring

**Group^a^**	**Lambing^b ^(Ewe ID)**	**PLACENTAE**		**PROGENY**							
			
		**No**.	**qRT-PCR** **(No. copies)**	**ID**	**Condition**	**Autolysis**	**Ab ELISA^e^**	**qRT-PCR blood (No. copies/ml blood)^f^**	**qRT-PCR tissues (Mean No. copies)**	**No. tissues POS/tissues tested**	**Tissue with highest RNA copy number**
	
A	T (77781)	1	Neg (0)	1	Foetus	+	Neg	Neg	Pos (63.9)	3/6	Thyroid
(108)				2	Foetus	+	Neg	Neg	Pos (52.8)	2/4	Kidney
				3	Foetus	+	Neg	Neg	Pos (86.1)	3/7	Kidney
	S (86348)	1	Pos^c^	4	Live lamb	-	Neg	Pos (360597.6)	Pos (36830.4)	10/10	Thyroid
	D (87632)	2	Pos^c^	5	Live lamb	-	Neg	Pos (1015.6)	Pos (2771.6)	5/10	Kidney
			Pos^c^	6	Live lamb	-	Pos	Neg (0.0)	Neg (0)	0/10	--
	D (89395)	1	NT	7	Live lamb	-	Neg	Pos (1421.9)	Neg (0)	0/10	--
				8	Live lamb	-	Pos	Pos (595.1)	Pos (133.8)	4/10	Skin
	S (82327)	1	Pos (1075.6)	9	Live lamb	-	CA	Pos (1016.9)	Pos (16.2)	3/11	Mesencephalon
B	T (87650)	1	Pos (5911.5)	10	Stillborn	-	Neg	Pos	Pos (21102.2)	7/7	Skin
(76)				11	Stillborn	+	Neg	Pos	Pos (69652.4)	8/8	Kidney
				12	Live lamb	-	Neg	Pos (1871601.6)	Pos (53731.6)	8/9	cerebellum
	T (77776)	2	Pos (71826.0)	13	Stillborn	-	Pos	Pos (9932.0)	Pos (23811.9)	10/10	Lymph node
			Pos (103529.6)	14	Stillborn	-	Pos	Pos (1225.3)	Neg (0)	0/10	--
				15	Stillborn	-	Pos	Pos (10230.2)	Pos (348.0)	5/10	Skin
	D (87630)	1	Pos (24294.6)	16	Live lamb	-	Pos	Neg (0.0)	Neg (0)	0/10	--
				17	Live lamb	-	Pos	Pos (2033.5)	Neg (0)	0/10	--
	S (86330)	1	Pos (2380.1)	18	Live lamb	-	CA	Pos (1129.8)	Pos (195.0)	1/11	Kidney
	D (77765)	1	Pos (2496.7)	19	Live lamb	-	Neg	Pos (1964.0)	Neg (0)	0/10	--
				20	Live lamb	-	Neg	Pos (1279244.8)	Pos (61151.4)	9/10	Cortex
C	S (77770)	1	Pos (53502.4)	21	Live lamb	-	Neg	Pos (73004.4)	Pos (59714.0)	10/10	Cortex
(55)	S (82314)	1	Pos (8196.0)	22	Live lamb	-	CA	Pos (1044864.0)	Pos (225808.4)	9/9	Cortex
	T (82338)	2	Pos (39392.4)	23	Live lamb	-	Neg	Pos (414482.4)	Pos (65253.0)	10/10	Thymus
				24	Stillborn	-	Neg	Pos (368889.6)	Pos (103645.6)	10/10	Skin
			Neg (0)^d^	25	Stillborn	+	Neg	Pos	Pos (32014.7)	8/8	Skin

NT, Not tested; CA, Colostral antibodies

^a ^In brackets, day of gestation when pestivirus was inoculated

^b ^T, triplet; D, double; S, single

^c ^Three placentas could not be assigned to the corresponding ewe; estimates of viral genome copy numbers were 820.1, 45176.6 and 11604.94

^d ^Partial inhibition

^e ^As previously reported[7]

^f ^Number of copies per ml blood from live lambs or cardiac blood from stillborns; quantitative data not provided for degraded cardiac blood or in the case of foetal fluids

Six animals (2 in group A and 4 in group B) were negative in all 10 tissues tested, though only 2 of them (Nos. 6 and 16) were also negative in blood (Table 1). On the opposite, 9 animals (1 from group A, 3 from group B and all 5 in group C) were positive in all the tissues tested (between 8 and 10) as well as in blood.

**Figure 3:**
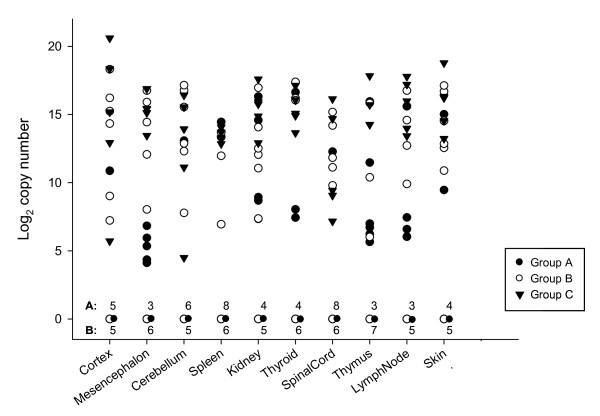
**Scatter Plot representing log_2 _viral genome copies in the different tissues from lambs, stillborns and fetuses**. Closed dots represent animals born to ewes in Group A; open dots, group B; and, triangles, group C. Number of negative animals in each tissue within groups A and B are indicated at the bottom of the graph.

Differences in viral genome copies were more marked in tissues than in blood. Taking into account the positive animals (at least one positive tissue), differences were observed among groups (*P *< 0.05), with significantly higher viral RNA loads in group C. Animals from group A had significantly lower viral RNA loads in every tissue analysed compared to those in group C. The only exception was lamb No. 4, born to a ewe from group A, which was positive in blood and all 10 tissues, with particularly high viral RNA loads in spleen, thymus, thyroid and kidney. No differences were observed between animals from groups B and C. Mean tissue genome copy numbers and organs with highest viral RNA load are indicated for each lamb in Table 1. Overall, cerebral cortex and skin were the tissues with the highest mean viral RNA loads, whereas spleen and spinal cord showed the lowest (Fig. 3). Skin, along with cerebral cortex and thyroid, were the tissues that consistently showed the highest viral RNA loads among animals positive in all tissues. No differences in viral genome copy numbers were observed when comparing stillborns and live lambs from the same group.

Fifteen placenta samples were collected, though 3 from group A could not be assigned to the corresponding ewe and another one was not analysed. Two placentae were negative, though partial inhibition was observed, and the remaining were positive (Table 1). It was noteworthy the case of two double pregnancies: one ewe that gave birth to 2 tissue-negative lambs (No. 16 and 17, the latter weakly positive in blood) despite the high positivity detected in the placenta, and another, that resulted in a lamb negative in all tissues but weakly positive in blood, and a heavily positive twin (Lambs Nos. 19 and 20, respectively) (Table 1).

Although in general twins and triplets behaved similarly with regard to virus distribution and quantity, important differences were observed in three cases (Table 1). Hence, a ewe from group A produced two lambs in two different placentas, a fully negative lamb with ELISA antibodies (No. 6), and another lamb without antibodies and RNA positive in blood and 5 tissues (No. 5), with particularly high viral genome copy numbers in kidney. In group B, it was noteworthy the case of two ewes. One produced 3 stillbirths in two placentas, with antibodies and RNA-positive blood, but clear differences in tissues, i.e., one stillborn (No. 14) was fully negative, another (No. 15) was weakly positive in 5 tissues, and another (No. 13) tested positive in all tissues. Finally, Lamb No. 19 (no antibodies, negative in all tissues, 1,964 copies/ml blood) and Lamb No. 20 (no antibodies, 9/10 tissues positive, 1.279,245 copies/ml blood) were born from the same placenta to the same ewe (group B), the ewe with the longest viraemia.

### Usefulness of qRT-PCR to discriminate between persistent infection and transient high viraemia

Considering all the data available (i.e. serology and viral genome copy number estimates in blood and tissues), we assessed the usefulness of qRT-PCR to discriminate between a persistent infection and a transient viraemia using single-time point blood sampling. Classifying as transient viraemic animals those with non-colostral antibodies or without antibodies but low levels of viral RNA in blood (foetal fluids or degraded cardiac blood excluded) and tissues (i.e., lambs No. 5-9 in group A and 13-19 in group B, Table 1), we established a quantitative cut-off threshold. This threshold, calculated as the arithmetic mean of the viral genome copy number per ml of blood estimated for these transient viraemic animals (χ) plus three times the standard deviation (χ + 3SD), was set at 13,275 copies. Values below this threshold would identify transient viraemic lambs. The threshold is clearly below the mean viral genome copy number per ml of blood observed for animals in group C (χ = 475,310). Using this threshold, lambs No. 12 and 20 (group B) and lamb No. 4 (group A) would be considered PI animals.

## Discussion

Research on diagnosis and control of ruminant pestiviruses has mainly focused on cattle, whereas studies in sheep are scarce. Antigen-ELISA has been successfully used to investigate the presence of PI animals in sheep flocks [10] but when virus load is low (transient viraemia), RT-PCR has shown better sensitivity [7]. Real time RT-PCR procedures for BDV detection have also been developed [11,12], but primers and probes showed certain mismatches with sequences from the Spanish strains. In the current study, we have tested a panpestivirus real-time quantitative RT-PCR (qRT-PCR) that also amplifies BDV genotype-4, which at present is the only genotype detected in Spanish sheep flocks [13-15]. In addition, the qRT-PCR used herein demonstrated a significant improvement in sensitivity compared to the results obtained with conventional RT-PCR on the same group of samples [8]. However, PCR does not distinguish between infectious and non-infectious virus and if sensitivity is very high, RT-PCR can detect pestivirus RNA also in transiently infected animals, which becomes a problem when the purpose is to identify PI animals. These problems are minimised when real-time RT-PCR is used for quantification and quantitative values are considered as proposed here.

Also important was the inclusion in this qRT-PCR procedure of primers and a probe for a host encoded gene which acted as an indicator of RNA integrity, since samples available for routine laboratory analysis of abortions are in many cases affected by different degrees of autolysis that can compromise RNA integrity. This internal control would also exclude false negative results caused by PCR inhibitors or loss of RNA during the extraction step. In fact, the amplification of the internal control confirmed that autolysis did not preclude viral RNA detection in tissues from the foetuses or the two stillborns that presented a certain degree of autolysis. In any case, a small underestimation of the real viral RNA load cannot be excluded, as was observed in two placentae. Finally, the quantitative results obtained in this study provided new information on the dynamics of the infection with local strains of BDV-genotype 4.

Pathogenesis of BDV is governed by various factors associated with the immunological status of the ewe and the age of the foetus at the time of exposure to the virus [16]. Infection of pregnant ewes with BDV during early or mid gestation results in abortion, stillbirths or unviable lambs, while PI animals are thought to be the result of infection during early embryonic and foetal development. Foetal immune competence is crucial to overcome the infection and it usually develops between days 60 and 80 of gestation. Following the criteria described by Nettleton and Willoughby [16], lambs from groups B or C (infection before day 80 of gestation) seronegative, with high viraemia and presence of the virus in most of the tissues would be considered as PI animals. This was the case for all the animals born to ewes in group C, which were all seronegative and specific viral RNA was detected in their blood and in all the tissues tested, generally at high levels. In group B, two lambs (Lambs No. 12 and 20) and two stillborns (No. 10 and 11) would be considered PI animals following those criteria. In any case, definitive confirmation would have required a second analysis a couple of weeks apart.

More difficult to interpret were the results found in lambs from group A, where most of them should have shown antibodies and no viraemia [16]. However, only 25% (2/8) of the animals had antibodies, and viral RNA was detected in blood or tissues of all except one, highlighting the sensitivity of qRT-PCR. Especially interesting was the case of lamb No. 4, a seronegative and healthy lamb [7] born to a ewe infected on day 108 of gestation. This animal presented high levels of viraemia, similar to lambs from group C. As mentioned above, to establish the PI status of this lamb, the analysis of a second blood sample taken two weeks after birth is necessary, but unfortunately the animal was sacrificed just after being born. Although with the available data we cannot categorically conclude if this was a highly viraemic new born lamb that had not developed an immune response or a PI, the quantitative data of viral genome copies in blood was clearly above the established threshold for transient viraemia. This would be indicative of persistent infection, suggesting that PI animals can also result from infections at all phases of gestation. On the other hand, a delay in immunocompetence has been suggested for goats [17], and in fact, seroprevalence in lambs from group A (inoculated at day 108 of gestation) was low, probably attributable to a slower seroconversion process as has been observed by other authors [18]. Conversely, detection of BDV in the presence of serum antibodies (lambs No. 8, 13, 15 and 17) has also been previously described [19,20] and this could be explained by the inability of animals to clear infection when virus content in the tissues is too high. As far as we know, the birth of PI lambs as a result of pestivirus infections after 80 days of gestation has not yet been reported, though most experimental infection studies challenge sheep at earlier stages of gestation [21-23]. A recent research article that included experimental infection of pregnant ewes at late gestation (days 120-125) with BVDV-2, reported clearance of BVDV from foetal tissues of ewes sacrificed at different time-points p.i. and the birth of three healthy, seropositive and virus-negative lambs from three ewes that were allowed to carry pregnancy to term [24]. However, virus isolation and not RT-PCR was used, and blood from the live lambs was the only sample tested.

In the study herein, it is also noteworthy the case of double and triple pregnancies that produced lambs with different virus distribution with instances where only one of two twin foetuses was virus-positive. This has been previously reported both in natural [25] and experimental infections [21,22] of sheep with pestivirus. These results suggest that BDV does not necessarily infect all lambs in uterus and show that the amount of virus invading the individual foetal tissues of twins varies.

Comparison of viral RNA loads in the different tissues offers the possibility of selecting the best samples for detection of BDV in the laboratory. Skin, brain (cerebral cortex), kidney and thyroid gland appeared to be the most reliable tissues for detecting the highest viral RNA loads. In a previous work, thyroid gland and kidney also gave the highest percentages of positivity by conventional RT-PCR, together with lymph nodes [8]. Conversely to results shown here, conventional RT-PCR had failed to detect pestiviral RNA in skin samples from animals positive in other tissues [8]. In the present study placenta was confirmed as an interesting sample to include in laboratorial protocols for diagnosis of BDV infection. Swasdipan et al. showed that pestivirus first establishes infection within the allantoic and amniotic membranes before reaching the foetus [22]. BDV replicates in the placentomes and has been regularly isolated from placental tissues [26]. The value of placenta in pestiviral diagnosis was also observed in sheep by other authors [24] who found a prolonged virus replication in placentomes from ewes inoculated at days 55-60 of gestation. Especially relevant are the results reported in goats where pestivirus antigen was most commonly detected within placenta than in other foetal tissues [27].

## Conclusion

The qRT-PCR tested here showed to be a rapid and highly sensitive method for the detection and quantification of pestiviruses in blood and different types of ovine tissues. Quantitative detection by qRT-PCR allowed us to monitor the BDV infection dynamics in experimentally infected sheep and study the distribution of viral RNA genome loads in different tissues from their offspring in relation with the moment of infection during gestation. Viral RNA quantification also proved to be an additional powerful tool for diagnosis and monitoring pestivirus infection in sheep. Although further studies are needed, questions regarding foetal immuno system development and the occurrence of persistent infections were raised.

## Competing interests

Vacunek S.L., the company manufacturing the qRT-PCR kit used in this study, is a spin-off from NEIKER and the authors from NEIKER are holders of a symbolic share in an organization linked with Vacunek.

## Authors' contributions

AH participated in the molecular design and the coordination of the study and drafted the manuscript. IS participated in the molecular design and carried out the molecular analyses. FB participated in the molecular design and critically revised the manuscript. EM participated in the experimental infection and critically revised the manuscript. RAJ performed the statistical analyses and participated in the critical reading of the publication. ALG conceived of the study and participated in its design and coordination, and helped to draft the manuscript.
